# A novel non-canonical Notch signaling regulates expression of synaptic vesicle proteins in excitatory neurons

**DOI:** 10.1038/srep23969

**Published:** 2016-04-04

**Authors:** Yukari Hayashi, Hiroshi Nishimune, Katsuto Hozumi, Yumiko Saga, Akihiro Harada, Michisuke Yuzaki, Takeshi Iwatsubo, Raphael Kopan, Taisuke Tomita

**Affiliations:** 1 Laboratory of Neuropathology and Neuroscience, Graduate School of Pharmaceutical Sciences, The University of Tokyo, Tokyo 113-0033, Japan; 2 Department of Physiology, School of Medicine, Keio University, Tokyo 160-8582, Japan; 3 Department of Anatomy and Cell Biology, University of Kansas Medical School, Kansas City, KS 66160, USA; 4 Department of Immunology, Tokai University School of Medicine, Kanagawa 259-1193, Japan; 5 Division of Mammalian Development, National Institute of Genetics, Shizuoka 411-8540, Japan; 6 Department of Genetics, SOKENDAI, Shizuoka 411-8540, Japan; 7 Department of Cell Biology, Graduate School of Medicine, Osaka University, Osaka 565-0871, Japan; 8 Department of Neuropathology, Graduate School of Medicine, The University of Tokyo, Tokyo 113-0033, Japan; 9 Department of Developmental Biology, Washington University School of Medicine, St. Louis, MO 63110, USA; 10 Division of Developmental Biology, Cincinnati Children’s Hospital Medical Center, University of Cincinnati College of Medicine, Cincinnati, OH 45229, USA

## Abstract

Notch signaling plays crucial roles for cellular differentiation during development through γ-secretase-dependent intramembrane proteolysis followed by transcription of target genes. Although recent studies implicate that Notch regulates synaptic plasticity or cognitive performance, the molecular mechanism how Notch works in mature neurons remains uncertain. Here we demonstrate that a novel Notch signaling is involved in expression of synaptic proteins in postmitotic neurons. Levels of several synaptic vesicle proteins including synaptophysin 1 and VGLUT1 were increased when neurons were cocultured with Notch ligands-expressing NIH3T3 cells. Neuron-specific deletion of Notch genes decreased these proteins, suggesting that Notch signaling maintains the expression of synaptic vesicle proteins in a cell-autonomous manner. Unexpectedly, cGMP-dependent protein kinase (PKG) inhibitor, but not γ-secretase inhibitor, abolished the elevation of synaptic vesicle proteins, suggesting that generation of Notch intracellular domain is dispensable for this function. These data uncover a ligand-dependent, but γ-secretase-independent, non-canonical Notch signaling involved in presynaptic protein expression in postmitotic neurons.

Notch receptors are highly conserved single-pass transmembrane proteins^1^,^2^ and play essential roles in proliferation and cell fate determination of progenitor cells during the development of central nervous system^3^,^4^. Notch is activated when binding of transmembrane DSL ligands (Delta-like (Dll) 1, Dll4, Jagged1 and Jagged2) expressed on a neighboring cell triggers unfolding and subsequent sequential proteolysis of the receptor. The mature Notch receptor exists as a heterodimer of proteolytically cleaved extracellular Notch (ECN) and transmembrane and intracellular (TMIC) domain. The ligand binding to ECN triggers the S2 cleavage in the juxtamembrane region of the TMIC to generate NEXT (for Notch extracellular truncation). NEXT undergoes intramembrane S3 cleavage, which is executed by PS/γ-secretase to release the Notch intracellular domain (NICD)^5^. This sequence is become known as the canonical Notch pathway in which NICD is the signal transducer, translocating into the nucleus to bind a DNA binding partner RBPjκ and activate transcription of target genes^6^.

Notch and its ligands are also expressed in adult brains^7^,^8^, albeit at a lower level. Notch signaling inhibits and enhances the outgrowth and branching, respectively, in neuronal cultures^9^,^10^,^11^,^12^,^13^, and is implicated in the synaptic plasticity *in vivo*^14^,^15^,^16^,^17^,^18^. Notably, CamKII-Cre-driven conditional deletion of Notch1 in mature neurons showed defects in long-term synaptic plasticity and short-term memory^8^. However, molecular basis of Notch signaling in the mature nervous system remains elusive. In addition, neurodegeneration that was caused by conditional ablation of γ-secretase in mature brains were not observed in conditional Notch- or RBPjκ-null mice^19^,^20^,^21^,^22^. These observations raise a possibility that an alternative Notch signaling which requires neither RBPjκ nor γ-secretase mediates synaptic functions or plasticity in mammalian neurons.

Here we examined the mechanism of mammalian Notch signaling in the postmitotic neurons. We found that activation of the endogenous Notch receptor with the DSL ligands in cultured neurons increased the expression levels of several synaptic vesicle proteins such as synaptophysin 1 and VGLUT1. Stimulus-dependent recycling of functional presynaptic terminals was also upregulated in the co-cultured neurons. Unexpectedly, γ-secretase activity is not required for the DSL ligand-dependent increment of synaptic vesicle proteins, suggesting that the canonical Notch signaling is not involved in these effects. Finally, we found that protein kinase G inhibitors abolished this Notch signaling. Our findings indicate that a novel, non-canonical Notch signaling may contribute to the synaptic formation or function in neurons.

## Results

### Notch proteins in postmitotic neurons

**Figure 1:**
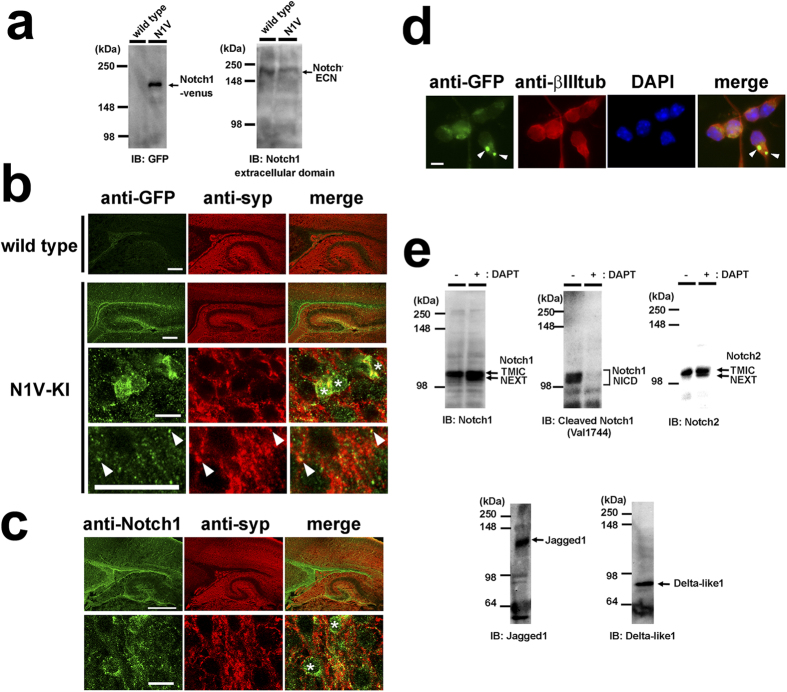
The expression of Notch and Notch ligands in mouse brain and primary neuronal culture. (**a**) Immunoblot analysis of Notch1-Venus protein in N1V-KI mouse brain. Brains of wild type or homozygous N1V-KI mice at postnatal day (P) 1 were homogenized and analyzed by immunoblotting using antibodies against GFP (left, TMIC of Notch1-venus) and Notch1 extracellular domain (right, ECN). (**b**) Representative images of sagittal sections of cerebral cortex from P1 wild type mouse or P0 homozygous N1V-KI mouse immunostained with anti-GFP (green) and anti-synaptophysin 1 (syp, red) antibodies. The bottom two panels of N1V-KI show a magnified image of cerebral cortical region. Asterisks indicate Notch1-positive cell bodies. Arrowheads indicate N1V-positive puncta colocalized with synaptophysin 1. Scale bars, 200 μm (the top two panels) and 10 μm (the bottom two panels). (**c**) Representative images of sagittal sections of cerebral cortex from wild type mouse at P0 immunostained for Notch1 intracellular domain (green) and synaptophysin 1 (red). Scale bar, 200 μm. The bottom panels are a magnified image of cerebral cortex. Scale bar, 10 μm. Asterisks indicate Notch1-positive cell bodies. (**d**) Expression of Notch1-Venus protein in 2 DIV cultured cortical neurons obtained from homozygous N1V-KI mouse immunostained with antibodies against GFP (green), neuron-specific β-III tubulin (red). Nuclei are co-stained with DAPI (blue). Notch1-Venus-positive puncta in soma are shown by arrowheads. Scale bar, 10 μm. (**e**) Immunoblot (IB) analyses of Notch1, Notch2, Jagged1 and Delta-like1/4 in 6 DIV primary neuronal culture obtained from wild type brains. Note that treatment with the γ-secretase inhibitor DAPT caused accumulation of NEXT of Notch1 and Notch2 and diminished the Notch1 NICD production.

We first characterized the expression pattern of Notch receptors in postmitotic neurons. To gain the detection sensitivity of immunostaining, we took advantage of Notch1-Venus knock-in (N1V-KI) mouse, which expresses chimeric Notch1 polypeptide fused with Venus protein at most C terminus of Notch1 under the endogenous Notch1 promoter. Homozygous N1V-KI mice were fertile and showed no gross developmental defect, suggesting that C-terminal fusion of Venus protein did not affect the Notch1 signaling *in vivo*. Immunoblot analysis of cerebral cortices from neonatal N1V-KI mice using antibodies against GFP or ECN showed expected size of bands, suggesting that N1V proteins were successfully expressed (Fig. 1a). Then we performed immunohistochemical analysis of cerebral cortical slices from NIV-KI and wild type mice. Venus immunoreactivity was predominantly observed in the subventricular zone and the hippocampal dentate gyrus of neonatal N1V-KI mouse brain, which was not detected in wild type tissue (Fig. 1b). We found an antibody (EP1238Y, Novus Biologicals) against the intracellular domain of Notch1 showed a similar staining pattern in the brain of wild type mouse (Fig. 1c). These results indicate that Notch1 protein is highly expressed in the subventricular zone and the hippocampal dentate gyrus. In addition, punctate immunoreactivity of Venus (in N1V-KI) or Notch1 proteins was present within the cell body of a fraction of cortical neurons (asterisks, Fig. 1b,c). Double-immunostaining with the presynaptic marker synaptophysin 1 revealed that some of N1V-positive puncta in cerebral cortex colocalized with synaptophysin 1, suggesting that Notch1 at least partially localized at synapses (arrowheads, Fig. 1b).

We next examined the expression pattern of Notch receptors in dissociated cortical neurons from N1V-KI mice. At 2 days *in vitro* (2 DIV), strong expression of Notch1-Venus protein was observed at the cell body of postmitotic neurons (Fig. 1d). Also, we detected the endogenous Notch1 TMIC and NICD in wild type neuronal culture by immunoblot analysis (Fig. 1e). In addition, Notch2 TMIC and DSL ligands (Jagged1 and Dll1/4) were detected as well. Treatment with γ-secretase inhibitor DAPT caused accumulation of Notch1/Notch2 NEXT polypeptides and disappearance of the Notch1 NICD. Taken together, these results suggest that Notch proteins are expressed in postmitotic neurons, and are constitutively activated by ligands in the neuronal culture.

### Notch ligand-expressing NIH3T3 cells increased the levels of synaptic vesicle proteins

**Figure 2:**
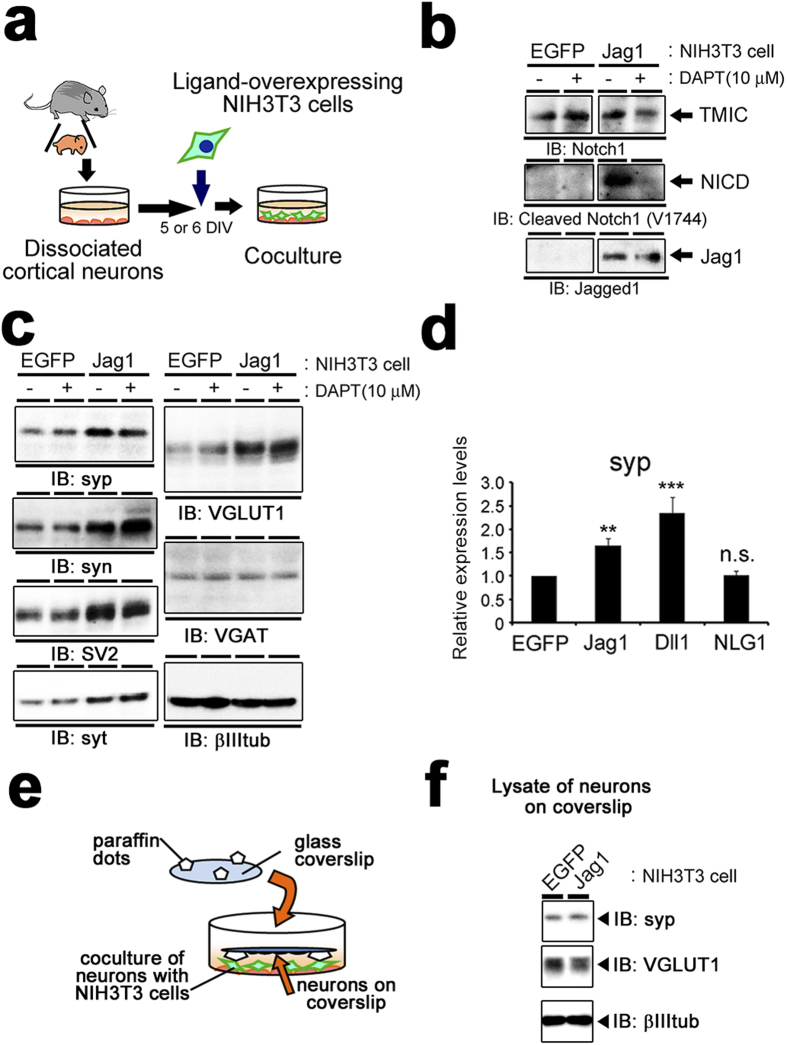
Coculture of neurons with Notch ligand-overexpressing cells. (**a**) Schematic depiction of the coculture of neurons and NIH3T3 cells. 5 or 6 DIV neurons were cocultured with Notch ligand-expressing NIH3T3 cells for 24 hours and subjected to the analyses. (**b**) Immunoblot analysis of primary neurons cocultured with NIH3T3 cells expressing either EGFP or Jagged1 with antibodies against Notch1 intracellular domain (upper), cleaved Notch1 (middle) and Jagged1 (bottom). The gels have been run under the same experimental conditions, and representative blots are cropped and used in this figure. Full-length gels are presented in Supplementary Fig. S1. (**c**) Immunoblot analysis of the cocultured neurons with antibodies against several synaptic membrane proteins. syp, synaptophysin 1; syn, synapsin 1; syt, synaptotagmin 1; βIIItub, neuron-specific β-III-tubulin. The gels have been run under the same experimental conditions, and representative blots are cropped and used in this figure. (**d**) Densitometric analysis of synaptophysin 1 levels in neurons cocultured with NIH3T3 cells expressing EGFP, Jagged1 (Jag1), Delta-like1 (Dll1) or Neuroligin 1 (NLG1). The band intensities of synaptophysin 1 were normalized to β-III-tubulin for each sample (n = 5–17, means ± SEM, **P < 0.01, ***P < 0.001, compared with EGFP by Steel-Dwass test). (**e**) Schematic depiction of co-culture without direct cell-cell contact where neurons on the coverslips and neuron cocultured with NIH3T3 cells at the bottom of culture dish. (**f**) Immunoblot analysis of lysates of neurons on coverslip shown in (**e**). Jag1-3T3 did not affect the protein levels of synaptophysin 1 and VGLUT1 in neurons on the coverslips, which do not interact with NIH3T3 cells in the same conditioned media of the coculture. The gels have been run under the same experimental conditions, and representative blots are cropped and used in this figure.

To examine the role of Notch signaling in postmitotic neurons, we aimed to activate Notch receptor by ligands employing coculture assay^23^. Jagged1 is considered to be one of the physiological ligands for Notch receptor at synapse^8^. We thus prepared Jagged1-IRES-EGFP-overexpressing NIH3T3 cells (Jag1-3T3) and cocultured them with cortical neurons at 5 or 6 DIV (Fig. 2a). Immunoblot analysis showed that the generation of Notch1 NICD was increased in neuronal culture when cocultured with Jag1-3T3 but not with EGFP-expressing 3T3 cells (EGFP-3T3) (Fig. 2b), confirming that canonical Notch signaling was activated in neuronal culture by interaction with Jag1-3T3. In addition, we found that coculture with Jag1-3T3 dramatically increased the levels of several synaptic vesicle membrane proteins, including synaptophysin 1, synapsin 1, SV2 and synaptotagmin 1 (Fig. 2c,d). By contrast, the levels of neuron-specific β-III-tubulin did not show any change. Intriguingly, Jag1-3T3 increased the protein levels of vesicular glutamate transporter 1 (VGLUT1), but not vesicular GABA transporter (VGAT). These results suggest that Jag1 upregulates the expression levels of synaptic vesicle membrane proteins exclusively in glutamatergic but not GABAergic neurons. NIH3T3 cells which overexpress Delta-like1 (Dll1-3T3) significantly increased the levels of synaptophysin 1 as well (Fig. 2d), suggesting that this effect is common to DSL ligands and not restricted to a specific ligand/receptor pair. Notably however, DAPT treatment did not abolish this augmentation (see below). We further tested the coculture with NIH3T3 cells overexpressing Neuroligin 1 (NLG1), a adhesion molecule^23^ which is involved in synapse differentiation. Unlike Notch ligands, NLG1 failed to affect the amount of synaptophysin 1 protein in coculture assay (Fig. 2d). Furthermore, we cultured neurons and Jag1-3T3 without direct cell-cell contact in the same culture medium. In contrast to the mixed coculture, Jag1-3T3 failed to increase synaptic vesicle protein (Fig. 2e,f), suggesting that the direct interaction of Notch with its ligands is responsible for increase in the presynaptic vesicle proteins.

**Figure 3:**
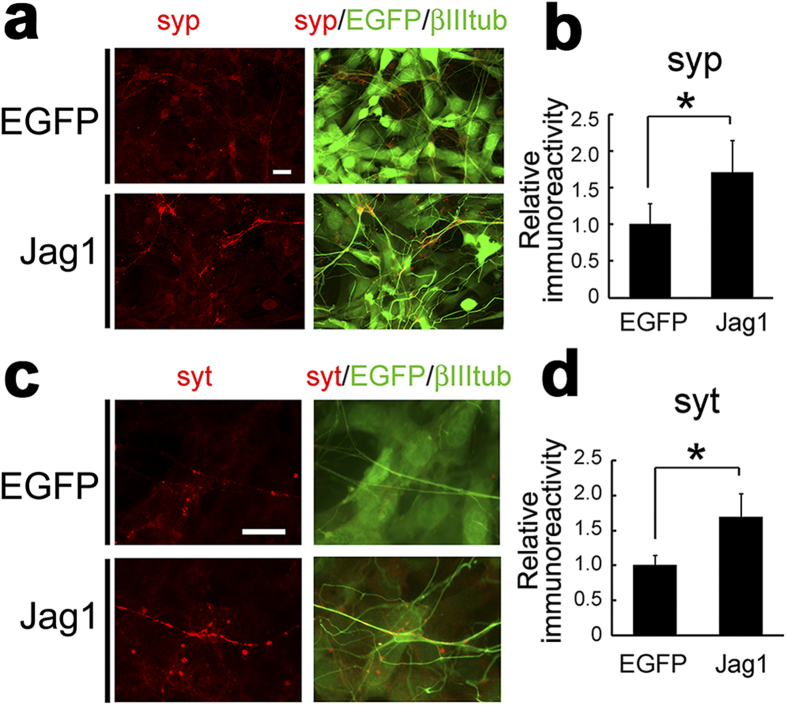
Induction of functional presynaptic terminals by Jagged1. (**a**) Primary cortical neurons cocultured with EGFP-3T3 or Jag1-3T3 were immunostained with antibodies against synaptophysin 1 (red) and β-III-tubulin (green). Note that Jag1-3T3 cells also express EGFP by IRES-mediated translation (green). (**b**) Quantification of immunofluorescence signals of synaptophysin 1 (n = 4, means ± SEM. *p < 0.05 by student’s *t*-test). (**c**) Immunostaining of internalized antibody against synaptotagmin 1 (red) after depolarization in cocultured primary neurons. (**d**) Quantification of immunofluorescence signals of synaptotagmin 1 (n = 5, means ± SEM. *p < 0.05 by student’s *t*-test). Scale bars, 10 μm.

Next, we performed immunocytochemical analysis of neurons cocultured with Jag1-3T3 or EGFP-3T3 cells. Punctate immunostaining by anti-synaptophysin 1 antibody was significantly increased on neurons cocultured with Jag1-3T3 relative to EGFP-3T3 (Fig. 3a,b). During the synaptogenesis, accumulation of synaptic vesicles at presynaptic active zones is the final process in the functional synapse formation^24^. To explore whether the increased synaptic vesicle proteins contributes to presynaptic functions, we visualized the recycling synaptic vesicles by high potassium-induced internalization of antibodies against the lumenal domain of synaptotagmin 1^25^. High potassium stimulation induces exocytosis of the synaptic vesicles, and anti-synaptotagmin 1 antibodies are incorporated in the functional presynaptic terminals (Supplementary Fig. S2). Immunofluorescence of internalized antibodies was observed more frequently and intensely in the axons of coculture with Jag1-3T3 cells than EGFP-3T3 cells (Fig. 3c,d). These data suggest that Jag1-induced increase in synaptic vesicle proteins may reinforce functional presynaptic formation.

### Neural Notch regulates synaptic vesicle protein expression

**Figure 4:**
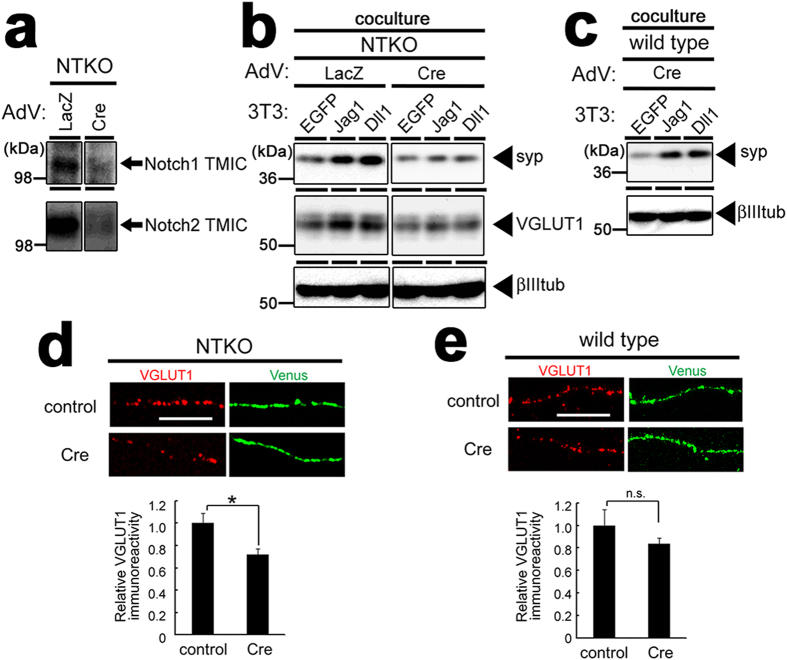
Notch proteins are indispensable to DSL ligand-induced increase in synaptic vesicle proteins. (**a**) Immunoblot analysis of neuronal culture from NTKO (*Notch1*^*flox/flox*^*; Notch2*^*flox/flox*^*; Notch3*^*−/−*^) mice. Infection of the recombinant adenovirus (AdV) encoding Cre recombinase (Cre) reduced the protein levels of Notch1 and 2 compared to that in cells infected with control adenovirus (LacZ). The gels have been run under the same experimental conditions, and representative blots are cropped and used in this figure. Full-length gels are presented in Supplementary Fig. S3. (**b**) Immunoblot analysis of AdV-infected neurons cocultured with NIH3T3 cells. The increase in the levels of synaptophysin 1 (syp) and VGLUT1 by Jagged1 or Delta-like1 was abolished by the infection of Cre AdV. The gels have been run under the same experimental conditions, and representative blots are cropped and used in this figure. (**c**) Immunoblot analysis of wild type primary neuronal culture infected with Cre AdV were cocultured with NIH3T3 cells. (**d, e**) Hippocampal neurons from NTKO mice (**d**) or wild type mice (**e**) were transfected with myristoylated Venus (green) with or without Cre recombinase and fixed at 10 DIV. The intensities of VGLUT1 immunofluorescence (red) along axon of neurons overexpressing Venus were quantified and shown below the panels (n = 5–10, means ± SEM, *p < 0.05 by student’s *t*-test). Scale bars, 5 μm.

To investigate whether the increase in synaptic vesicle proteins caused by Notch ligands requires their cognate Notch receptors, we utilized neuronal culture from NTKO mice, in which *Notch1* and *Notch2* genes were both floxed and Notch3 genes are deleted (*Notch1*^*flox/flox*^*;Notch2*^*flox/flox*^*;Notch3*^*−/−*^)^26^,^27^. The loci coding for Notch proteins was deleted following infection with recombinant adenovirus encoding Cre recombinase (Fig. 4a). The protein levels of synaptophysin 1 and VGLUT1 in Cre-expressed NTKO neuronal culture, but not wild type neurons, were no longer increased by coculture with Jag1- or Dll1-3T3 (Fig. 4b,c). Because Notch ligand expressing cells were still capable of increasing presynaptic protein levels in Notch3-deficient NTKO primary neurons infected with control adenovirus, this effect is mediated by Notch1 and/or Notch2. In addition, cultured hippocampal neurons from NTKO mice overexpressed with Cre recombinase contained significantly lower VGLUT1 levels along their axons (Fig. 4d), relative to wild type neurons (Fig. 4e). These results indicates that neuronal Notch1 and/or Notch2 regulate VGLUT1 protein levels in postmitotic hippocampal neurons.

**Figure 5:**
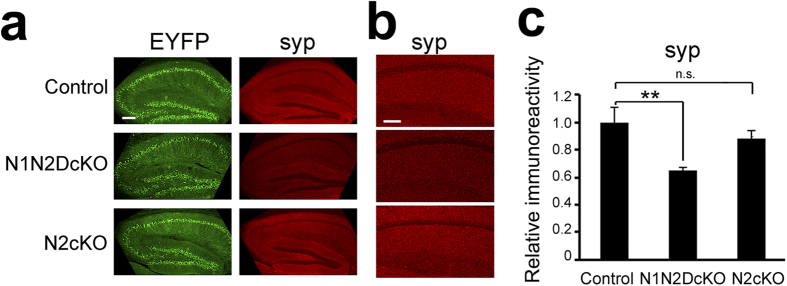
Levels of synaptophysin 1 in Notch conditional knockout mouse brains. (**a**) The representative hippocampal images of adult neuron-specific Notch1/Notch2 double-knockout mice (N1N2DcKO), *Notch2* knockout mice (N2cKO) and control mice. Sagittal sections of 23–28 week-old mice brains were immunostained for EYFP (green) and synaptophysin 1 (syp, red). Inactivation of *Notch1* and *Notch2* genes were achieved by endogenous tau promoter-driven CrePR protein. EYFP signals indicate neuronal cells with successful recombination at *Rosa26*^*stopfloxYFP*^ allele. Scale bar, 200 μm. (**b**) Magnified images of CA1 region in (**a**). Scale bar, 100 μm. (**c**) Quantitative analysis of synaptophysin 1 immunoreactivity. The immunofluorescence signals of CA1 were calculated (n = 4 mice each, means ± SEM, *p < 0.05 by Tukey’s test).

To assess the significance of these observations *in vivo*, we bred NTKO mice with *tau*^*CrePR*^ knock-in mice^28^ and *Gt(ROSA)26Sor*^*tm1(EYFP)Cos*^ EYFP mice^29^ to generate neuron-specific conditional *Notch1/Notch2* double-knockout mice (*Notch1*^*flox/flox*^*;Notch2*^*flox/flox*^*;Notch3+*^/−^*;tau*^*CrePR*^*;Rosa26*^*stopfloxEYFP*^ or N1N2DcKO), *Notch2* knockout mice (*Notch1*^*flox/*^*+;Notch2*^*flox/flox*^*;Notch3+*^*/−*^*;tau*^*CrePR*^*;Rosa26*^*stopfloxEYFP*^ or N2cKO) and control mice (*Notch1*^*flox/*^*+;Notch2*^*flox/*^*+;Notch3+*^*/−*^*;tau*^*CrePR*^*;Rosa26*^*stopfloxEYFP*^). As previously described, *tau*^*CrePR*^ mice produced a leaky Cre protein that acted in the absence of the agonist RU486^30^ in the brains of 23–28 week old mice (Fig. 5a)^31^. The efficiency of recombination was not improved by RU486 injection in our experiments (data not shown). We found that immunostaining for synaptophysin 1 produced a weak signal in N1N2DcKO mice compared with control mice or N2cKO mice (Fig. 5a). Synaptophysin 1 immunofluorescence intensity in the hippocampal CA1 region was significantly lower in the N1N2DcKO mice (Fig. 5b,c). These data suggest that either *Notch1* or both *Notch1* and *Notch2* are essential to maintain the expression of synaptophysin 1 *in vivo*. Taken all together, our data demonstrate that Notch acts cell-autonomously to regulate the expression of synaptic vesicle proteins in postmitotic neurons.

### γ-Secretase-independent non-canonical Notch signaling increases synaptic vesicle proteins

**Figure 6:**
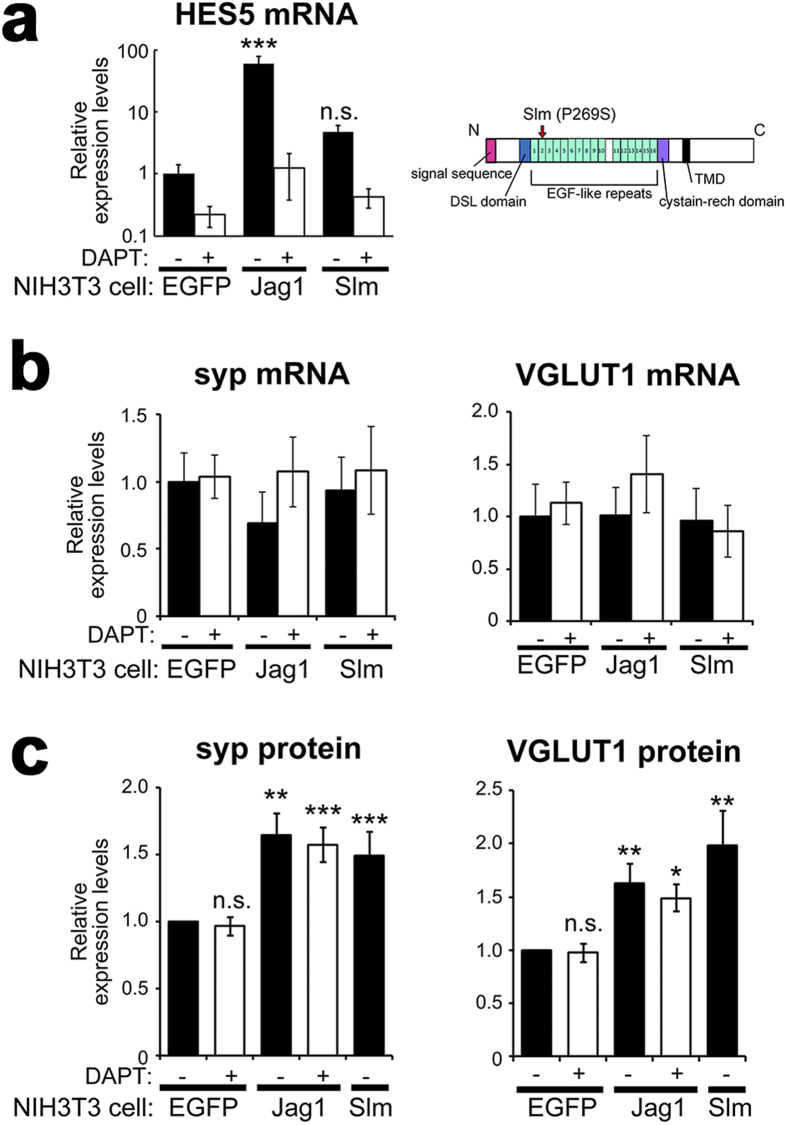
Non-canonical Notch signaling increased the levels of synaptic vesicle proteins. (**a**) Induction of Hes5 mRNA expression quantified using qRT-PCR analysis (n = 4–5, means ± SEM, **P < 0.01, ***P < 0.001, compared with EGFP control by Tukey’s test). Expressed proteins in NIH3T3 cells were indicated below the panels. DAPT was used at 10 μM. Schematic depiction of Slm mutation (P269S) in the murine Jagged1 at right. (**b**) Relative mRNA levels of synaptophysin 1 (syp) and VGLUT1 in the cocultures. (**c**) Relative protein expression levels of synaptophysin 1 (syp) and VGLUT1 quantified from the band intensities in immunoblot analyses of the coculture (n = 5–17, means ± SEM, *P < 0.05, **P < 0.01, ***P < 0.001, compared with EGFP control by Steel-Dwass test).

In canonical Notch pathway, ligand-induced Notch endoproteolysis by γ-secretase generates NICD, which transduces the Notch signal. However, DAPT^32^, a γ-secretase inhibitor, did not affect Notch ligands-dependent elevation of synaptic vesicle proteins (Fig. 2c). To elucidate the necessity of the NICD-dependent canonical signaling, we quantified mRNA levels of a *Notch* target gene, *Hes5*^33^, which is expressed in the neuronal culture. Quantitative real time PCR analysis (qRT-PCR) revealed that transcription of *Hes5* was upregulated in neuronal cultures cocultured with Jag1-3T3 and blocked by addition of 10 μM DAPT (Fig. 6a). In contrast, mRNA levels of synaptophysin 1 or VGLUT1 were unchanged in cocultures with Jag1-3T3 (Fig. 6b). Moreover, the elevated protein levels of synaptophysin 1 and VGLUT1 by Jag1-3T3 were induced even in the presence of DAPT (Figs 2c and 6c), indicating that the increase in presynaptic proteins by Jag1/Notch interaction was not the consequence of NICD-dependent transcription. To investigate this mechanism further, we employed a mutant Jagged1, *Slalom* (Slm). Slm is a mutation in *Jagged1* with a single amino acid substitution (P269S) that was originally identified in mutant mice with abnormalities in the patterning of the organ of Corti in the inner ear^34^, similar to *headturner* mutant mouse^35^, suggesting that Slm represents a loss-of-function allele of *Jagged1* (Fig. 6a). Consistent with this notion, Jagged1^Slm^ failed to upregulate *Hes5* transcription in cocultured neurons (Fig. 6b). Aforementioned results that DAPT did not affect Jagged1 function raised the possibility that Slm mutation might still be capable of elevating synaptic vesicle protein levels despite loss of ability to activate canonical Notch signaling. In agreement with this hypothesis, the levels of synaptophysin 1 and VGLUT1 were still augmented in neurons cocultured with Jagged1^Slm^-expressing NIH3T3 cells (Fig. 6c). These data indicate that Notch-ligand interaction-dependent, but NICD-independent, Notch signaling increased the expression levels of synaptic vesicle proteins.

**Figure 7:**
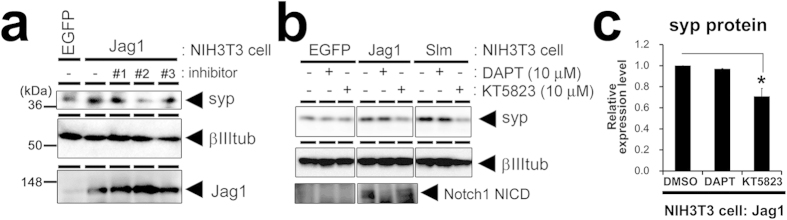
PKG inhibitor repressed Jagged1-induced increase in synaptophysin 1. (**a**) Representative immnoblot images of rat primary neuronal culture cocultured with NIH3T3 cells for synaptophysin 1 (syp), β-III-tubulin (βIIItub) and Jagged1 (Jag1). Coculture was maintained for 18 hours with or without several inhibitors: Rp-cAMPS triethylamine (#1), H-9 dihydrochloride (#2) and Ro 20-1724 (#3) (representative data from 4 experiments). Jag1-induced increase in the levels of synaptophysin 1 was blocked by H-9 dihydrochloride. (**b**) Representative immunoblot images for synaptophysin 1 (syp), β-III-tubulin (βIIItub) and Notch1 NICD in the coculture of murine primary neuronal culture and NIH3T3 cells. DAPT or KT5823 is treated at 10 μM. The gels have been run under the same experimental conditions, and representative blots are cropped and used in this figure. Full-length gels are presented in Supplementary Fig. S4. (**c**) Relative protein levels of synaptophysin 1 (syp) quantified from the immunoblot analyses of the coculture with Jag1-expression NIH3T3 cells (n = 4, means ± SEM, *P < 0.05 by Steel-Dwass test).

Finally, we addressed the question what is the non-canonical signal mechanism that underlies the increase in synaptic vesicle proteins by screening of several known enzyme inhibitors. Among them, H-9 dihydrochloride, an inhibitor of cAMP- and cGMP-dependent protein kinase (PKA and PKG, respectively), is found to attenuate the Jagged1-induced increase in synaptophysin 1 protein without affecting Jagged1 expression (Fig. 7a). We further tested a more selective inhibitor against PKG (KT5823, 10 μM), which abolished the synaptophysin 1 increment induced by Jagged1 as well as Jagged1^Slm^ (Fig. 7b,c). NICD generation by Jagged1 was not blocked by KT5823 (Fig. 7b), suggesting that KT5823 did not compromise Jag1-Notch interaction or γ-secretase-dependent signaling. Collectively, these findings suggest that PKG activity is required for Notch-dependent increase of the synaptic vesicle proteins.

## Discussion

In this study, we found that DSL ligand-induced stimulation upregulated the levels of several synaptic vesicle proteins in cultured neurons. Deletion of both *Notch1* and *Notch2* diminished the expression of VGLUT1, suggesting that Notch receptors regulate the expression of the presynaptic proteins in the glutamatergic neurons in a cell-autonomous manner. Loss of synaptophysin 1 in hippocampal CA1 area of neuron-specific N1N2 double KO mice but not of N2 cKO mice pointed out the possibility that Notch1 is responsible for synaptic formation, while Notch2 deletion is tolerated. Intriguingly, the Notch-dependent increment of synaptic vesicle proteins was insensitive to the treatment of γ-secretase inhibitor and thus did not require NICD release. Furthermore, a mutant Jagged1 that failed to activate NICD signaling retained the ability to increase synaptic vesicle proteins. Finally, our data suggest that a PKG-dependent pathway is the candidate downstream mechanism of this non-canonical Notch signaling.

Several lines of evidences suggested that Notch signaling has important roles not only in development, but in synaptic functions of brains. Synthetic peptide-induced activation of Notch signaling has been shown to facilitate the formation of LTP^36^, suggesting that ligand-Notch interaction modulates synaptic responses. CamKII-Cre-driven deletion of Notch1 in excitatory neurons disrupted synaptic plasticity in hippocampus and memory acquisition^8^. However, the molecular mechanism underlying the Notch signaling which modulates synaptic functions remains largely uncertain. Our study for the first time demonstrated that neuronal Notch is implicated in expression of essential proteins for presynaptic assembly.

A previous report demonstrated that most of the Notch mRNAs in adult brains derived from the cells other than excitatory neurons^19^. In contrast, we detected neuronal Notch1 proteins both in immature neurons of the dissociated culture and the neonatal brains *in vivo*, which might indicate that Notch protein is more enriched in young neurons. Moreover, our findings implicate a role for non-canonical Notch signaling in postmitotic neurons, which is consistent with a previous study showing that impaired cognitive performance was not observed in RBPjκ-cKO mice^22^, indicating a possibility that neurons may prefer the γ-secretase/NICD-independent non-canonical Notch signaling rather than canonical pathway for cognitive function. However, we also detected NICD in our coculture assay. A previous study reported that neuronal activity generates NICD in Arc-dependent manner in hippocampal neurons^8^. Additionally, it is shown that overexpression of NICD in pyramidal neurons in the visual cortex resulted in reduced spine density and LTP^37^. These observations suggest that the NICD-dependent signaling may also play a certain role in neurons. How non-canonical and canonical pathways are controlled in neurons for synaptic function would be determined by future studies.

To date, certain non-canonical Notch signaling has been reported, while no common pathway was identified^38^,^39^,^40^. There had been no study that examined a non-canonical pathway in mammalian nervous system. We found that PKG inhibition can block the increase of synaptophysin 1 induced by ligands without affecting NICD generation, suggesting that PKG activity is required as a downstream factor for this non-canonical Notch signaling. PKG is a kinase that is activated by cGMP in the nitric oxide (NO) pathway and harbors critical function in long term memory formation^41^. Notably, it was reported that PKG activity correlates with the presynaptic function^42^ as well as expression of presynaptic membrane proteins^43^, the latter being an important role in the fear memory consolidation. In this model, retrograde NO signaling from the postsynaptic sites to activate PKG signaling in the presynaptic terminal is critical for the increased production of synaptic vesicle protein and homeostatic control of neurotransmission. Several studies have been reported that Notch signaling interacts with NO/PKG pathway in non-neuronal cells through distinct mechanisms^44^,^45^,^46^. Our study provides evidence for a novel mode of signal crosstalk between NICD-independent Notch signaling with PKG pathway in neurons. Since the neuronal non-canonical Notch signaling does not require receptor proteolysis, ligand-Notch interaction might trigger NO/PKG signaling via protein-protein interaction with intracellular domain of Notch receptor just beneath the plasma membrane. Notch and its ligand interaction might function as a local cue to the retrograde NO signaling in neurons, or loss of NO signaling may be epistatic to signal activation. Our observations are reminiscent chronic treatment with brain-derived neurotrophic factor (BDNF), which also increased the levels of specific synaptic vesicle proteins^47^,^48^. Thus it would be intriguing to test whether PKG inhibition affects chronic BNDF effect and if either participates in non-canonical Notch signaling at the presynaptic membrane.

Recently many synaptic cell-adhesion molecules including NLGs have been identified as synaptic organizers^49^, which trans-synaptically interact with their ligands or receptors and induce differentiation or functional modulation of synapses. Coculture with NLG1-overexpressed non-neuronal cells is known to induce formation of presynaptic properties in the contacting axons. In the present study, we described that Notch ligand augmented the levels of the synaptic vesicle proteins. Since mRNA levels were not affected, non-canonical Notch signaling might regulate either translation or protein stabilization of these synaptic molecules. In contrast, NLG1 did not have this effect, suggesting that NLGs accumulate presynaptic proteins rather than increase the expression of them. Interestingly, however, deletion of NLGs *in vivo* resulted in impaired synaptic transmission as well as decreased levels of several synaptic proteins including synaptophysin 1, synaptotagmin 1 and VGLUT1, although synaptogenesis was not disturbed^50^, suggesting that chronic loss of NLG functions lead destabilization of synaptic vesicle proteins. On the other hand, non-canonical Notch signaling regulates synaptic vesicle protein expression, which might augment the functional synaptic vesicles and affect synaptic formation and transmission^51^. Or otherwise, Notch may cooperatively function with NLGs/neurexins or other synaptic cell-adhesion molecule in modulation of synaptic function. Many synaptic adhesion molecules including NLGs are associated with cognitive disorder^52^,^53^. Involvement of disturbed Notch signaling in cognitive disorders had been implicated by the evidences that mental retardation is associated with some cases of Alagille syndrome, which is caused by loss-of-function-mutation in *JAGGED1* gene^54^. Our study shed light on a novel mode of DSL ligand-dependent Notch signaling, giving a new insight for understanding the mechanism for neural Notch signaling and its contributions on synaptic or cognitive functions.

## Materials and Methods

### Animals

All experimental procedures were performed in accordance with the guidelines for animal experiments of the University of Tokyo, were approved by the Institutional Animal Care and Use Committees (IACUC)/ethics committee of the Graduate School of Pharmaceutical Sciences, the University of Tokyo (Protocol No. P25-3). N1V-KI mice were originally generated at National Institute of Genetics, Japan. Briefly, a gene encoding green fluorescence Venus protein and floxed PGK-neo cassette was inserted into mouse *Notch1* locus to fuse the Venus at most C terminus of Notch1 polypeptide. ES cells were selected by G418 resistance and injected into the blastocyst. Heterozygous knock-in mouse was obtained from chimeric mouse and crossed with CAG-Cre mouse to excise PGK-neo cassette region. Homozygous N1V-KI mice are viable and fertile with no observed gross developmental defects. NTKO mice described previously^27^ were maintained as homozygous lines. *Tau*^*CrePR*^ knock-in mice^28^ and *Gt(ROSA)26Sor*^*tm1(EYFP)Cos*^ EYFP reporter mice^29^ (The Jackson Laboratory) were crossed and maintained as *tau*^*CrePR/CrePR*^*;Rosa26*^*stopfloxEYFP/stopfloxEYFP*^ double reporter homozygous mice. NTKO mice and double reporter mice were crossed to generate quintuple heterozygous parents, and finally N1N2DcKO, N2cKO and control mice used in Fig. 6 were obtained.

### Plasmids

Retroviral vectors encoding Jagged1-IRES-EGFP and Delta-like1-IRES-EGFP were reported previously^55^. The Jagged1 Slm mutant vector was generated by long PCR-based protocol using following primer pairs: 5′-AAG TGC ATC CCG CAC TCA GGA TGT GTC CAC GGT ACC TGC AAT GAA-3′ as a sense primer and 5′-TTC ATT GCA GGT ACC GTG GAC ACA TCC TGA GTG CGG GAT GCA CTT-3′ as an antisense primer. A cDNA encoding HA-NLG1^23^ was gift from Dr. Peter Scheiffele (University of Basel, Switzerland) and inserted into pMIGR1^56^. pCAG-Cre:GFP was purchased from Addgene (#13776). pCX-Myristoylated Venus^57^ vector was gifted from Dr. Anna-Katerina Hadjantonakis (Sloan-Kettering Institute).

### Cell culture, retroviral and adenoviral infection and transfection

Primary dissociated cortical neuronal cultures were prepared from mice at embryonic day (E) 16-17 or E18 Wistar rat and maintained in Neurobasal medium (Invitrogen) supplied with B27 supplement (Invitrogen) as previously described^56^., Dissociated cells were plated on poly-l-ornithine (SIGMA)-coated plastic plates or glass coverslips at 2.5 × 10^5^ cells/cm^2^ for immunoblot analysis, and at 1.25 × 10^5^ cells/cm^2^ for immunocytochemistry. At 4 DIV, 100 μM D-AP5 (SIGMA) was added. For coculture with NIH3T3 cells, NIH3T3 cells was added at the density of 1.25 × 10^5^ cells/cm^2^ (for immunoblot analysis) or 6.25 × 10^5^ cells/cm^2^ (for immunocytochemistry) at 5 or 6 DIV. Except for Fig. 7, 10 μM DAPT^32^ was added 24 hours before the coculture with NIH3T3 cells. For Fig. 7, inhibitors were treated 40 min after adding NIH3T3 cells. Enzyme Inhibitor LIGAND-SET, Rp-cAMPS triethylamine, H-9 dihydrochloride, Ro 20-1724 and KT5823 were purchased from SIGMA. For hippocampal culture, cells are dissociated from E16-18 hippocampi of wild type or NTKO mice and plated at 4000 cells/cm^2^ on glass coverslips coated with poly-l-ornithine. The coverslips were cultured in the astrocyte culture dish. Astrocytes were prepared from P1 wild type Balb/C mice cortices. For Fig. 4d,e, neurons were transfected using LipofectAMINE2000 (Invitrogen) at 5 or 6 DIV then treated with 100 μM D-AP5. For Fig. 4a–c, adenoviral infection was performed at 2 DIV by adding diluted (1:20 in culture medium) adenoviral stock solutions at 100 μl/cm^2^. After incubating for 1 hour, fresh medium were added by 250 μl/cm^2^. Retroviral infection into NIH3T3 cells were performed as previously described^58^.

### Visualization of recycling synaptic vesicles by antibody uptake

Cultures were incubated at 37 °C for 8 min with isotonic Tyrode high K solution (20 mM HEPES pH7.2–7.5, 64 mM NaCl, 90 mM KCl, 2 mM MgCl_2_, 10 mM glucose, 2 mM CaCl_2_, 50 μM D-AP5 and 10 μM CNQX) containing rabbit anti-synaptotagmin 1 lumenal domain antibodies (1:200, #105002, synaptic systems). After incubation, cultures were washed with modified Tyrode solution (20 mM HEPES pH 7.2–7.5, 150 mM NaCl, 4 mM KCl, 2 mM MgCl_2_, 10 mM glucose), fixed and processed for immunocytochemistry.

### Adenovirus preparation

Plasmids for generation of recombinant adenovirus encoding LacZ or Cre recombinase were provided from Dr. Rita Balice-Gordon^59^ (The University of Pennsylvania). For large scale purification of adenoviruses, HEK293 cells was infected with adenoviral medium and cultured until cytopathic effect was observed. Adenoviral was purified from 150 ml medium using Vivapure Adenopack 100 (Vivascience) according to manufacturer’s instructions. The eluted virus were concentrated using vivaspin 20 (Vivascience) to ~5 × 10^12^ optical particle units/ml. Viral concentration was estimated by measuring OD260. Final tock solutions were preserved at −80 degree before use and never underwent freeze-and-thaw cycles.

### Quantitative Real-time PCR analysis

Total mRNAs were isolated and purified from cell cultures using ISOGEN reagent (Nippon Gene). 0.5 or 1 μg of purified mRNAs from each sample were reverse-transcribed into cDNA using Rever Tra Ace qPCR RT kit (TOYOBO). The 1 μl of cDNA templates were used for real-time PCR reaction and relative quantification using LightCycler 450 (Roche) with SYBR Green I protocol. The amount of *Hes5* mRNA was normalized to *Gapdh* mRNA in each sample. The amount of mRNAs of Synaptophysin 1 (*Syp*), VGLUT1 (*Slc17a*) and VGAT (*Slc32a1*) were normalized to the β-III-tubulin (*Tubb3*) mRNA. The following primer pairs were used to detect the indicated target genes: 5′-agtacccattcaggctgcac-3′ (forward) and 5′-ccgaggaggagtagtcacca-3′ (reverse) for *Syp*, 5′-cccactccatgtgagtcca-3′ (forward) and 5′- caacataaatacagaggtggctaaaa-3′ (reverse) for *Tubb3*, 5′-actgcaccccagcatctc-3′ (forward) and 5′- ccagggtgtgttaaacttcgt-3′ (reverse) for *Slc17a*, for 5′-atgccattcagggcatgt-3′ (forward) and 5′-ggcgaagatgatgaggaaca-3′ (reverse) *Slc32a1*, 5′-tggagatgctcagtcccaag-3′ (forward) and 5′-tgctctatgctgctgttgatg-3′ (reverse) for *Hes5* and 5′-ctgcaccaccaactgcttag-3′ (forward) and 5′-actgtggtcatgagcccttc-3′ (reverse) for *Gapdh*.

### Antibodies

Following antibodies were used: Notch1 (EP1238Y, NOVUS), intracellular domain of Notch1 (C-20, Santa Cruz), extracellular domain of Notch1 (#07-218, Upstate Biotechnology), Jagged1 (C-20, Santa Cruz), Delta-like1/4 (C-20, Santa Cruz), cleaved Notch1 (V1744) (#2412, Cell signaling technology), synaptophysin 1 (MAB5258, Chemicon), VGLUT1 (#135303, Synaptic Systems), VGAT (#131002, Synaptic Systems), Cre recombinase (#18771, IBL), GFP (A6455, Invitrogen), synapsin1 (#AB1543 Chemicon) and β-III-tubulin (TUJ1, SIGMA). Antibodies against synaptotagmin 1^60^ were gift from Dr. Masami Takahashi (Kitasato University). The monoclonal antibodies against Notch2 (C651.6DbHN) and SV2 were obtained from the Developmental Studies Hybridoma Bank developed under the auspices of the NICHD and maintained by The University of Iowa, Department of Biology, Iowa City, IA 52242.

### Immunoblot analysis and quantitation of immunoblot band densities

Total cells were lysed in 2% SDS, briefly sonicated and then incubated at 37 °C for 30 min with 1% 2-mercanptoethanol. After SDS-PAGE, samples were transferred to polyvinylidene difluoride membrane. The membranes were incubated in 5% Slim milk and 0.2% Tween-20, treated with primary antibodies and then probed with HRP-conjugated secondary antibodies (GE Healthcare) and chemiluminescent signals were acquired. Band intensities were measured using MultiGauge software (GE Healthcare) or ImageJ software (NIH). The ratio of synaptophysin 1 or VGLUT1 to β-III-tubulin were acquired and normalized to the value of control.

### Quantitative immunofluorescence analysis

For Fig. 3, immunostained images were acquired in a blind manner to experimental condition using fluorescence microscope. Using the ImageJ software, background was subtracted and the averaged immunofluorescent signals per total area were obtained and normalized to the mean values of control experiments. For Fig. 3c,d, non-specific signals were removed manually before analyses. For Fig. 4d,e, co-expression of Cre-GFP and Myristoylated Venus was confirmed by comparing the emission wavelength from somata (Cre-GFP) and distal neurites (Myristoylated Venus) using lambda scanning mode according to manufacturer’s instruction (TCS-SP5, Leica). The isolated axonal tracts which do not contact with other cells were selected, and the signal intensities of VGLUT1 immunofluorescence of z-stack images were normalized to the axonal length. For Fig. 5, z-stack images of hippocampi from each mouse were analyzed by ImageJ. The signals of synaptophysin 1 in hippocampal CA1 stratum radiatum were obtained by subtracting the signals in subventricular zone as background and compared.

### Immunocytochemisty

Cells were fixed with 4% paraformaldehyde/4% sucrose in PBS for 15–30 min, incubated in PBS containing 0.1% TritonX-100/3% BSA and stained with primary antibodies. The cells were washed and incubated for 1 h with Alexa Fluor-conjugated secondary antibodies (Invitrogen) and/or DAPI (WAKO Chemicals). The cells were washed and mounted and imaged with a fluorescence microscope (Axio observer Z1, Zeiss) or a confocal laser-scanning microscope (TCS-SP5, Leica).

### Immunohistochemistry

Newborn (P0-1) mice were killed by decapitation and the heads were fixed by soaking in PBS containing 4% paraformaldehyde overnight. The tissues were serially rinsed in PBS containing 10, 20, and 30% Sucrose, covered by OCT compound (Sakura Finetek Japan) and frozen in liquid nitrogen. Frozen sections (10 μm) were prepared using Cryostat. Adult mice were anesthetized and fixed by cardiac perfusion with PBS containing 4% paraformaldehyde. The brain was post-fixed at 4 °C overnight. Sagittal slices (100 μm) were prepared using a microslicer (DTK-2000; D.S.K.; Dosaka, Kyoto, Japan). Sections were blocked with PBS containing 10% cow serum. Adult samples were permeabilized with 0.2% Triton X-100. The sections were then incubated with primary antibodies overnight, followed by incubation with Alexa 546 or 647 conjugated secondary antibodies (Invitrogen) for 2 hrs. The samples were viewed using a confocal laser-scanning microscope (TCS-SP5, Leica).

### Statistical analysis

For analyses of immunofluorescence and qRT-PCR, student *t*-test was used for comparisons between two-group data and Tukey’s test was used for multiple group comparisons. For quantitative immunoblot analysis, Steel-Dwass test was used. In figures, statistical significance is indicated by (*) for p < 0.05, (**) for p < 0.01, and (***) for p < 0.001. All data are presented as mean ± SEM.

## Additional Information

**How to cite this article**: Hayashi, Y. *et al.* A novel non-canonical Notch signaling regulates expression of synaptic vesicle proteins in excitatory neurons. *Sci. Rep.* **6**, 23969; doi: 10.1038/srep23969 (2016).

## Supplementary Material

Supplementary Information

## References

[b1] Kopan R. Notch signaling. Cold Spring Harb Perspect Biol 4, 10.1101/cshperspect.a011213 (2012). PMC3475170 23028119

[b2] Hori K., Sen A. & Artavanis-Tsakonas S. Notch signaling at a glance. J Cell Sci 126, 2135–2140, 10.1242/jcs.127308 (2013). 23729744 PMC3672934

[b3] Ables J. L., Breunig J. J., Eisch A. J. & Rakic P. Not(ch) just development: Notch signalling in the adult brain. Nat Rev Neurosci 12, 269–283, 10.1038/nrn3024 (2011). 21505516 PMC3159580

[b4] Louvi A. & Artavanis-Tsakonas S. Notch signalling in vertebrate neural development. Nat Rev Neurosci 7, 93–102 (2006). 16429119 10.1038/nrn1847

[b5] Kopan R., Schroeter E. H., Weintraub H. & Nye J. S. Signal transduction by activated mNotch: importance of proteolytic processing and its regulation by the extracellular domain. Proc Natl Acad Sci USA 93, 1683–1688 (1996). 8643690 10.1073/pnas.93.4.1683 PMC40002

[b6] Schroeter E. H., Kisslinger J. A. & Kopan R. Notch-1 signalling requires ligand-induced proteolytic release of intracellular domain. Nature 393, 382–386 (1998). 9620803 10.1038/30756

[b7] Stump G. *et al.* Notch1 and its ligands Delta-like and Jagged are expressed and active in distinct cell populations in the postnatal mouse brain. Mech Dev 114, 153–159 (2002). 12175503 10.1016/s0925-4773(02)00043-6

[b8] Alberi L. *et al.* Activity-induced Notch signaling in neurons requires Arc/Arg3.1 and is essential for synaptic plasticity in hippocampal networks. Neuron 69, 437–444 (2011). 21315255 10.1016/j.neuron.2011.01.004 PMC3056341

[b9] Sestan N., Artavanis-Tsakonas S. & Rakic P. Contact-dependent inhibition of cortical neurite growth mediated by notch signaling. Science 286, 741–746 (1999). 10531053 10.1126/science.286.5440.741

[b10] Franklin J. L. *et al.* Autonomous and non-autonomous regulation of mammalian neurite development by Notch1 and Delta1. Curr Biol 9, 1448–1457 (1999). 10607588 10.1016/s0960-9822(00)80114-1

[b11] Berezovska O. *et al.* Notch1 inhibits neurite outgrowth in postmitotic primary neurons. Neuroscience 93, 433–439 (1999). 10465425 10.1016/s0306-4522(99)00157-8

[b12] Redmond L., Oh S. R., Hicks C., Weinmaster G. & Ghosh A. Nuclear Notch1 signaling and the regulation of dendritic development. Nat Neurosci 3, 30–40 (2000). 10607392 10.1038/71104

[b13] Breunig J. J., Silbereis J., Vaccarino F. M., Sestan N. & Rakic P. Notch regulates cell fate and dendrite morphology of newborn neurons in the postnatal dentate gyrus. Proc Natl Acad Sci USA 104, 20558–20563 (2007). 18077357 10.1073/pnas.0710156104 PMC2154470

[b14] Presente A., Boyles R. S., Serway C. N., de Belle J. S. & Andres A. J. Notch is required for long-term memory in Drosophila. Proc Natl Acad Sci USA 101, 1764–1768 (2004). 14752200 10.1073/pnas.0308259100 PMC341850

[b15] Ge X. *et al.* Notch signaling in Drosophila long-term memory formation. Proc Natl Acad Sci USA 101, 10172–10176 (2004). 15220476 10.1073/pnas.0403497101 PMC454384

[b16] Costa R. M., Honjo T. & Silva A. J. Learning and memory deficits in Notch mutant mice. Curr Biol 13, 1348–1354 (2003). 12906797 10.1016/s0960-9822(03)00492-5

[b17] Giniger E. Notch signaling and neural connectivity. Curr Opin Genet Dev 22, 339–346, 10.1016/j.gde.2012.04.003 (2012). 22608692 PMC3426662

[b18] Kidd S., Struhl G. & Lieber T. Notch is required in adult Drosophila sensory neurons for morphological and functional plasticity of the olfactory circuit. PLoS Genet 11, e1005244, 10.1371/journal.pgen.1005244 (2015). 26011623 PMC4444342

[b19] Zheng J. *et al.* Conditional deletion of Notch1 and Notch2 genes in excitatory neurons of postnatal forebrain does not cause neurodegeneration or reduction of Notch mRNAs and proteins. J Biol Chem 287, 20356–20368, 10.1074/jbc.M112.349738 (2012). 22505716 PMC3370217

[b20] Saura C. A. *et al.* Loss of presenilin function causes impairments of memory and synaptic plasticity followed by age-dependent neurodegeneration. Neuron 42, 23–36, 10.1016/S0896-6273(04)00182-5 (2004). 15066262

[b21] Tabuchi K., Chen G., Sudhof T. C. & Shen J. Conditional Forebrain Inactivation of Nicastrin Causes Progressive Memory Impairment and Age-Related Neurodegeneration. Journal of Neuroscience 29, 7290–7301, 10.1523/Jneurosci.1320-09.2009 (2009). 19494151 PMC2719251

[b22] Sato C. *et al.* Loss of RBPj in postnatal excitatory neurons does not cause neurodegeneration or memory impairments in aged mice. PLoS One 7, e48180, 10.1371/journal.pone.0048180 (2012). 23110206 PMC3482205

[b23] Scheiffele P., Fan J., Choih J., Fetter R. & Serafini T. Neuroligin expressed in nonneuronal cells triggers presynaptic development in contacting axons. Cell 101, 657–669 (2000). 10892652 10.1016/s0092-8674(00)80877-6

[b24] Sigrist S. J. & Schmitz D. Structural and functional plasticity of the cytoplasmic active zone. Curr Opin Neurobiol 21, 144–150, 10.1016/j.conb.2010.08.012 (2011). 20832284

[b25] Biederer T. & Scheiffele P. Mixed-culture assays for analyzing neuronal synapse formation. Nat Protoc 2, 670–676, 10.1038/nprot.2007.92 (2007). 17406629

[b26] Pan Y. *et al.* gamma-secretase functions through Notch signaling to maintain skin appendages but is not required for their patterning or initial morphogenesis. Developmental cell 7, 731–743, 10.1016/j.devcel.2004.09.014 (2004). 15525534

[b27] Bai S. *et al.* NOTCH1 regulates osteoclastogenesis directly in osteoclast precursors and indirectly via osteoblast lineage cells. J Biol Chem 283, 6509–6518, 10.1074/jbc.M707000200 (2008). 18156632

[b28] Hashimoto Y. *et al.* Neuron-specific and inducible recombination by Cre recombinase in the mouse. Neuroreport 19, 621–624, 10.1097/WNR.0b013e3282fb7d99 (2008). 18382274

[b29] Srinivas S. *et al.* Cre reporter strains produced by targeted insertion of EYFP and ECFP into the ROSA26 locus. BMC Dev Biol 1, 4 (2001). 11299042 10.1186/1471-213X-1-4 PMC31338

[b30] Kitayama K. *et al.* Purkinje cell-specific and inducible gene recombination system generated from C57BL/6 mouse ES cells. Biochem Biophys Res Commun 281, 1134–1140, 10.1006/bbrc.2001.4492 (2001). 11243853

[b31] Muramatsu K. *et al.* Neuron-specific recombination by Cre recombinase inserted into the murine tau locus. Biochem Biophys Res Commun 370, 419–423, 10.1016/j.bbrc.2008.03.103 (2008). 18381201

[b32] Morohashi Y. *et al.* C-terminal fragment of presenilin is the molecular target of a dipeptidic gamma-secretase-specific inhibitor DAPT (N-[N-(3,5-difluorophenacetyl)-L-alanyl]-S-phenylglycine t-butyl ester). J Biol Chem 281, 14670–14676, 10.1074/jbc.M513012200 (2006). 16569643

[b33] Ohtsuka T. *et al.* Hes1 and Hes5 as notch effectors in mammalian neuronal differentiation. EMBO J 18, 2196–2207, 10.1093/emboj/18.8.2196 (1999). 10205173 PMC1171303

[b34] Tsai H. *et al.* The mouse slalom mutant demonstrates a role for Jagged1 in neuroepithelial patterning in the organ of Corti. Hum Mol Genet 10, 507–512 (2001). 11181574 10.1093/hmg/10.5.507

[b35] Kiernan A. E. *et al.* The Notch ligand Jagged1 is required for inner ear sensory development. Proc Natl Acad Sci USA 98, 3873–3878, 10.1073/pnas.071496998 (2001). 11259677 PMC31145

[b36] Wang Y. *et al.* Involvement of Notch signaling in hippocampal synaptic plasticity. Proc Natl Acad Sci USA 101, 9458–9462 (2004). 15190179 10.1073/pnas.0308126101 PMC438998

[b37] Dahlhaus M. *et al.* Notch1 signaling in pyramidal neurons regulates synaptic connectivity and experience-dependent modifications of acuity in the visual cortex. J Neurosci 28, 10794–10802 (2008). 18945887 10.1523/JNEUROSCI.1348-08.2008 PMC6671381

[b38] Giniger E. A role for Abl in Notch signaling. Neuron 20, 667–681 (1998). 9581760 10.1016/s0896-6273(00)81007-7

[b39] Heitzler P. Biodiversity and noncanonical Notch signaling. Curr Top Dev Biol 92, 457–481, 10.1016/S0070-2153(10)92014-0 (2010). 20816404

[b40] Gentle M. E., Rose A., Bugeon L. & Dallman M. J. Noncanonical Notch signaling modulates cytokine responses of dendritic cells to inflammatory stimuli. J Immunol 189, 1274–1284, 10.4049/jimmunol.1103102 (2012). 22753939 PMC3442230

[b41] Giese K. P. & Mizuno K. The roles of protein kinases in learning and memory. Learn Mem 20, 540–552, 10.1101/lm.028449.112 (2013). 24042850

[b42] Eguchi K., Nakanishi S., Takagi H., Taoufiq Z. & Takahashi T. Maturation of a PKG-dependent retrograde mechanism for exoendocytic coupling of synaptic vesicles. Neuron 74, 517–529, 10.1016/j.neuron.2012.03.028 (2012). 22578503

[b43] Ota K. T., Monsey M. S., Wu M. S. & Schafe G. E. Synaptic plasticity and NO-cGMP-PKG signaling regulate pre- and postsynaptic alterations at rat lateral amygdala synapses following fear conditioning. PLos One 5, e11236, 10.1371/journal.pone.0011236 (2010). 20574537 PMC2888610

[b44] Charles N. *et al.* Perivascular nitric oxide activates notch signaling and promotes stem-like character in PDGF-induced glioma cells. Cell Stem Cell 6, 141–152, 10.1016/j.stem.2010.01.001 (2010). 20144787 PMC3818090

[b45] Chang A. C. *et al.* Notch initiates the endothelial-to-mesenchymal transition in the atrioventricular canal through autocrine activation of soluble guanylyl cyclase. Dev Cell 21, 288–300, 10.1016/j.devcel.2011.06.022 (2011). 21839921

[b46] El-Sehemy A. *et al.* Notch activation augments nitric oxide/soluble guanylyl cyclase signaling in immortalized ovarian surface epithelial cells and ovarian cancer cells. Cell Signal 25, 2780–2787, 10.1016/j.cellsig.2013.09.008 (2013). 24041655

[b47] Tartaglia N. *et al.* Protein synthesis-dependent and -independent regulation of hippocampal synapses by brain-derived neurotrophic factor. J Biol Chem 276, 37585–37593 (2001). 11483592 10.1074/jbc.M101683200

[b48] Yamada M. K. *et al.* Brain-derived neurotrophic factor promotes the maturation of GABAergic mechanisms in cultured hippocampal neurons. J Neurosci 22, 7580–7585 (2002). 12196581 10.1523/JNEUROSCI.22-17-07580.2002 PMC6757965

[b49] Siddiqui T. J. & Craig A. M. Synaptic organizing complexes. Curr Opin Neurobiol 21, 132–143, 10.1016/j.conb.2010.08.016 (2011). 20832286 PMC3016466

[b50] Varoqueaux F. *et al.* Neuroligins determine synapse maturation and function. Neuron 51, 741–754, 10.1016/j.neuron.2006.09.003 (2006). 16982420

[b51] Rosenberg T. *et al.* The roles of protein expression in synaptic plasticity and memory consolidation. Front Mol Neurosci 7, 86, 10.3389/fnmol.2014.00086 (2014). 25429258 PMC4228929

[b52] Singh S. K. & Eroglu C. Neuroligins provide molecular links between syndromic and nonsyndromic autism. Sci Signal 6, re4, 10.1126/scisignal.2004102 (2013). 23838185 PMC4000534

[b53] Sudhof T. C. Neuroligins and neurexins link synaptic function to cognitive disease. Nature 455, 903–911, 10.1038/nature07456 (2008). 18923512 PMC2673233

[b54] Oda T. *et al.* Mutations in the human Jagged1 gene are responsible for Alagille syndrome. Nature Genetics 16, 235–242, 10.1038/Ng0797-235 (1997). 9207787

[b55] Abe N., Hozumi K., Hirano K., Yagita H. & Habu S. Notch ligands transduce different magnitudes of signaling critical for determination of T-cell fate. Eur J Immunol 40, 2608–2617, 10.1002/eji.200940006 (2010). 20602435

[b56] Suzuki K. *et al.* Activity-dependent proteolytic cleavage of neuroligin-1. Neuron 76, 410–422, 10.1016/j.neuron.2012.10.003 (2012). 23083742

[b57] Rhee J. M. *et al.* *In vivo* imaging and differential localization of lipid-modified GFP-variant fusions in embryonic stem cells and mice. Genesis 44, 202–218, 10.1002/dvg.20203 (2006). 16604528 PMC2887760

[b58] Kitamura T. *et al.* Retrovirus-mediated gene transfer and expression cloning: powerful tools in functional genomics. Exp Hematol 31, 1007–1014 (2003). 14585362

[b59] Gonzalez M. *et al.* Disruption of Trkb-mediated signaling induces disassembly of postsynaptic receptor clusters at neuromuscular junctions. Neuron 24, 567–583 (1999). 10595510 10.1016/s0896-6273(00)81113-7

[b60] Takamori M., Hamada T., Komai K., Takahashi M. & Yoshida A. Synaptotagmin can cause an immune-mediated model of Lambert-Eaton myasthenic syndrome in rats. Ann Neurol 35, 74–80, 10.1002/ana.410350112 (1994). 8285596

