# Supplementary material for: A novel non-canonical Notch signaling regulates expression of synaptic vesicle proteins in excitatory neurons
Source: Sci Rep. 2016 Apr 4;6:23969. doi: 10.1038/srep23969 (PMC4819173; doi:10.1038/srep23969)
Supplement: Supplementary Information [file srep23969-s1.doc]

**Supplementary Information for**

**A novel non-canonical Notch signaling regulates expression of synaptic vesicle proteins in excitatory neurons**

Yukari Hayashi, Hiroshi Nishimune, Katsuto Hozumi, Yumiko Saga, Akihiro Harada, Michisuke Yuzaki, Takeshi Iwatsubo, Raphael Kopan, Taisuke Tomita*

*Laboratory of Neuropathology and Neuroscience, Graduate School of Pharmaceutical Sciences, The University of Tokyo, Tokyo 113-0033, Japan*

Corresponding author,

Dr. Taisuke Tomita

Laboratory of Neuropathology and Neuroscience,
Graduate School of Pharmaceutical Sciences,
The University of Tokyo, Tokyo 113-0033, Japan

Tel.: +81 3 5841 4868

Fax: +81 3 5841 4868

Email: taisuke@mol.f.u-tokyo.ac.jp


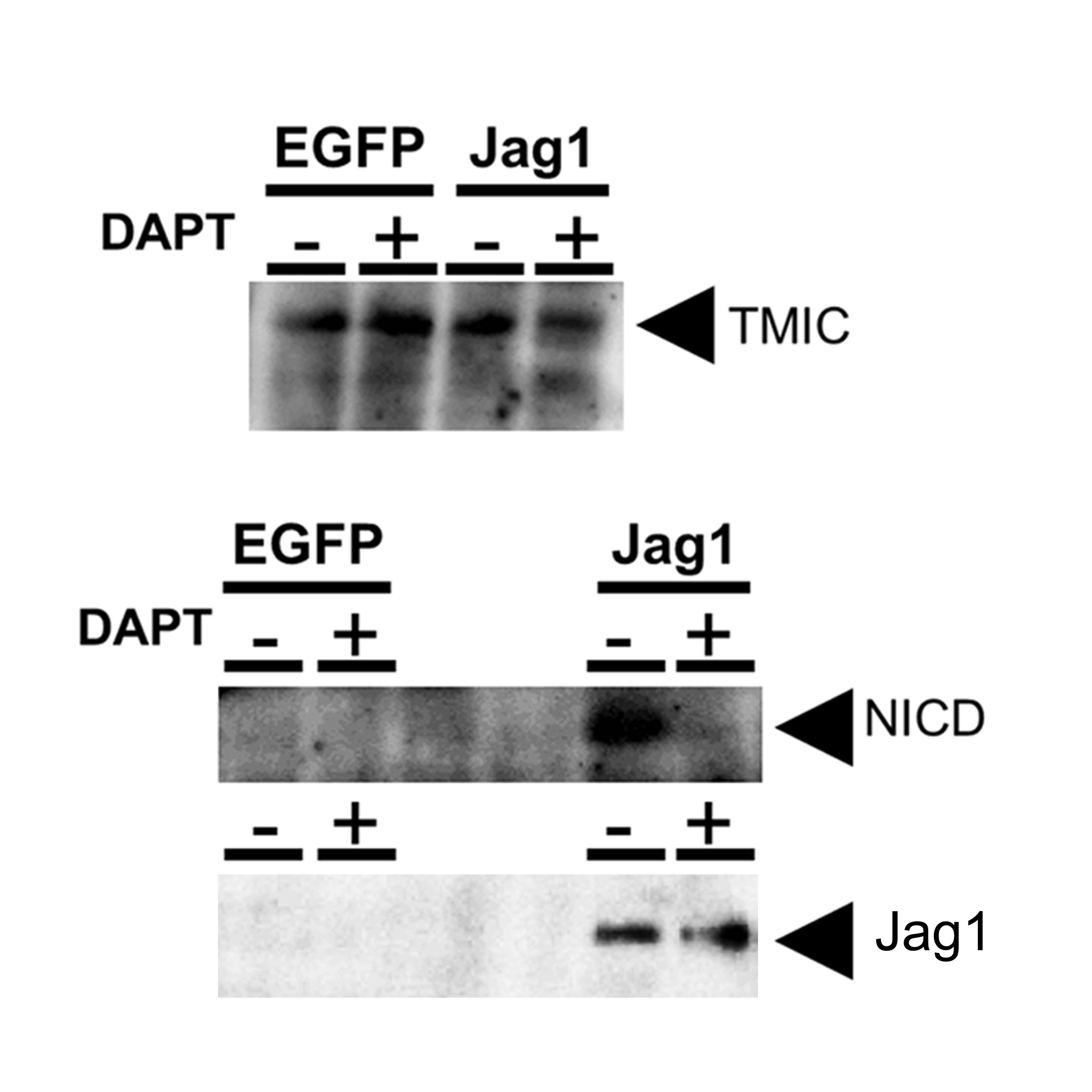


Supplemental Fig. S1. Uncropped immunoblot for Figure 2b


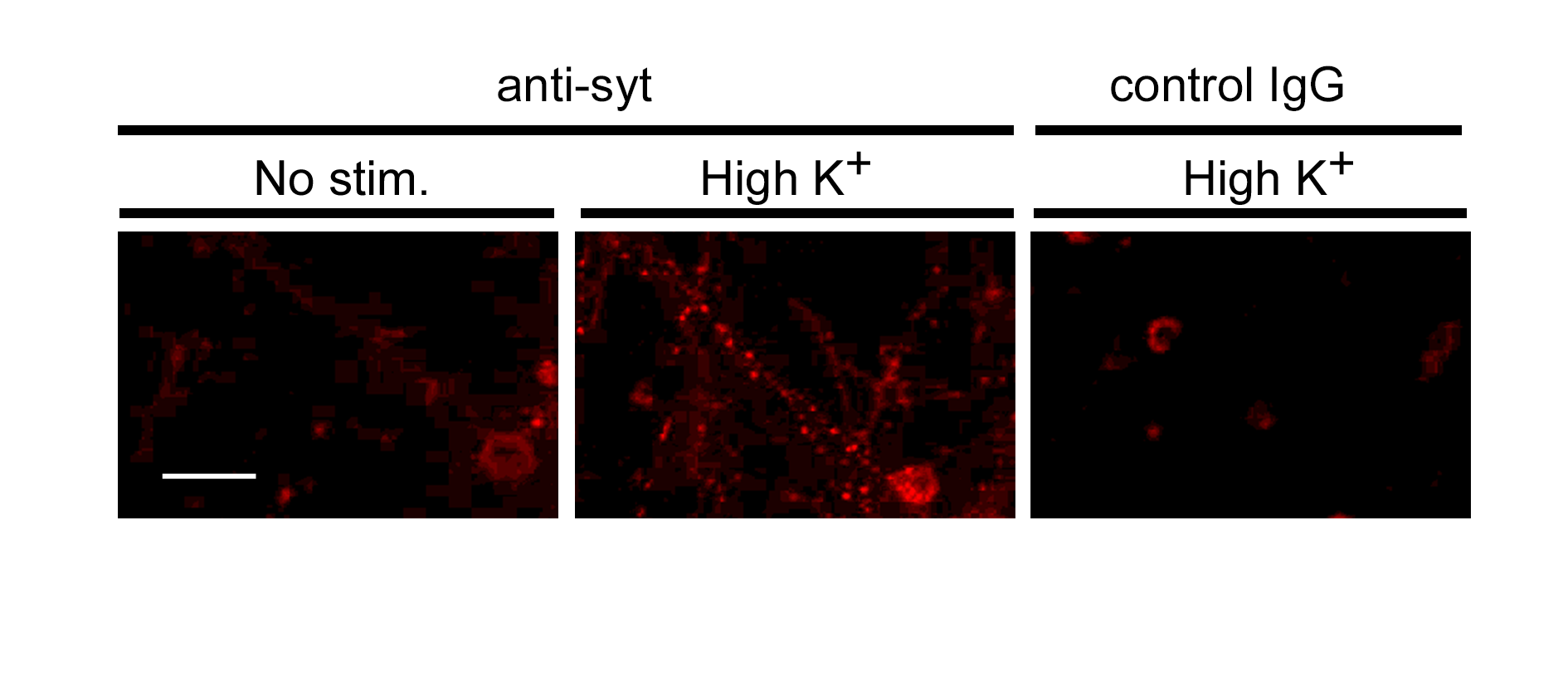


Supplemental Fig. S2. High-potassium-induced uptake of anti-synaptotagmin 1 antobody.

DIV8 primary cortical neurons are incubated for 5 min with normal Tyrode solution (No stim.) or isotonic Tyrode high K solution (High K+) with antibodies against synaptotagmin 1 lumenal domain (anti-syt) or control rabbit igG (Control IgG). Cells are fixed and stained with Alexa Fluor-conjugated anti-rabbit IgG antibodies (red). Punctate immunofluorescent signals of synaptotagmin 1 are increased by high potassium stimulation. Scale bar, 20 mm


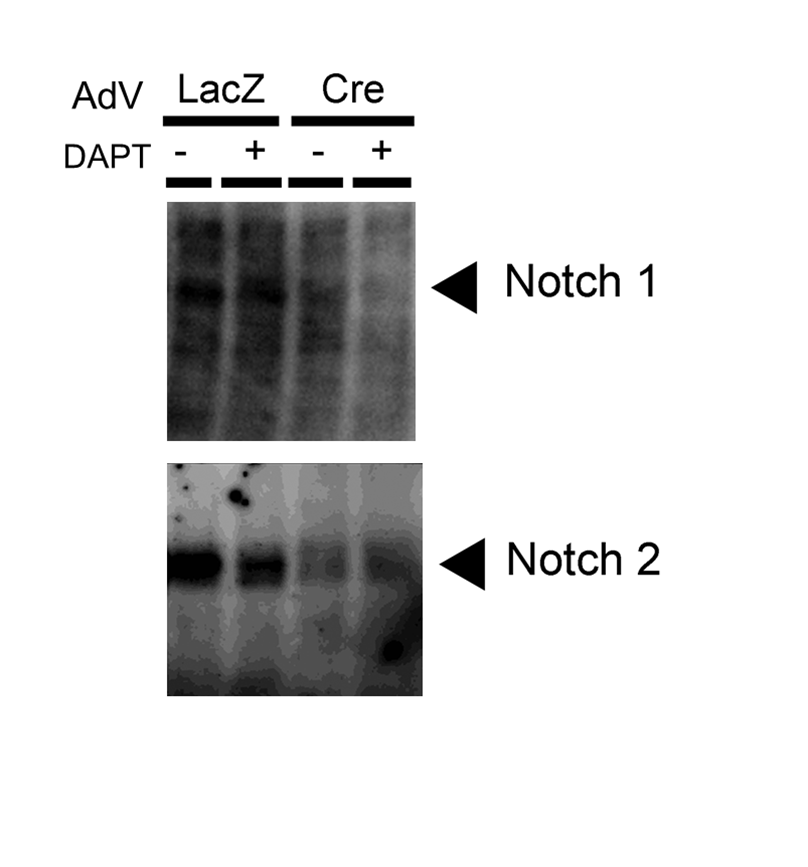


Supplemental Fig. S3. Uncropped immunoblot for Figure 4a


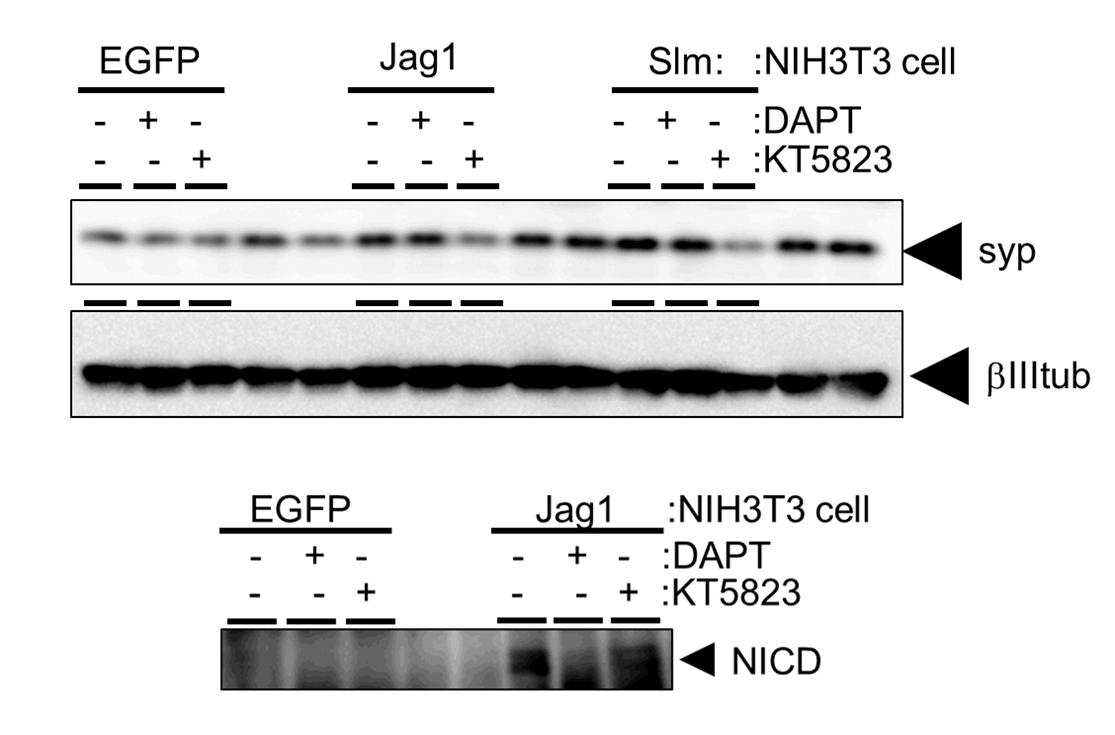


Supplemental Fig. S4. Uncropped immunoblot for Figure 7b and 7c
